# Distinct iron acquisition strategies in oceanic and coastal variants of the mixotrophic dinoflagellate *Karlodinium*

**DOI:** 10.1093/ismejo/wraf099

**Published:** 2025-05-20

**Authors:** Se Hyeon Jang, YuanYu Lin, Adrian Marchetti

**Affiliations:** Department of Earth, Marine and Environmental Sciences, University of North Carolina at Chapel Hill, Chapel Hill 27514, NC, United States; Department of Oceanography, Chonnam National University, Gwangju 61186, Republic of Korea; Department of Earth, Marine and Environmental Sciences, University of North Carolina at Chapel Hill, Chapel Hill 27514, NC, United States; Department of Earth, Marine and Environmental Sciences, University of North Carolina at Chapel Hill, Chapel Hill 27514, NC, United States

**Keywords:** dinoflagellates, iron-limited regions, iron acquisition, mixotrophy, oceanic and coastal variants, transcriptomics

## Abstract

The availability of the micronutrient iron is important in regulating phytoplankton growth across much of the world’s oceans, particularly in the high-nutrient, low-chlorophyll regions. Compared to known mechanisms of iron acquisition and conservation in autotrophic protists (e.g. diatoms), those of dinoflagellates remain unclear, despite their frequent presence in offshore iron-limited waters. Here, we investigate the strategies of an ecologically important mixotrophic dinoflagellate to coping with low iron conditions. Coupled gene expression and physiological responses as a function of iron availability were examined in oceanic and coastal strains of the dinoflagellate *Karlodinium*. Under iron-replete conditions, grazing was only detected in coastal variants, resulting in faster growth rates compared to when grown autotrophically. Under iron-limited conditions, all isolates exhibited slower growth rates, reduced photosynthetic efficiencies, and lower cellular iron quotas than in iron-replete conditions. However, oceanic isolates exhibited higher relative growth rates compared to coastal isolates under similar low iron concentrations, suggesting they are better adapted to coping under iron limitation. Yet the oceanic isolates did not exhibit the ability to appreciably reduce cell volume or increase iron-use efficiencies compared to the coastal isolates to cope with iron limitation, as often observed in oceanic diatoms. Rather, molecular pathway analysis and corresponding gene expression patterns suggest that oceanic *Karlodinium* utilizes a high-affinity iron uptake system when iron is low. Our findings reveal cellular mechanisms by which dinoflagellates have adapted to low iron conditions, further shedding light on how they potentially survive in variable iron regions of the world’s oceans.

## Introduction

Dinoflagellates (Alveolata) are one of the most ecologically important phytoplankton groups in marine ecosystems, playing critical roles in oxygen production, food webs, and carbon and nutrient cycles [1–3]. Members exhibit various life forms (i.e. free-living, parasitic, and mutualistic), life cycles (e.g. cyst formation), and even a multitude of nutritional modes: autotrophy, heterotrophy, or mixotrophy [2, 4, 5]. Particularly, many phototrophic dinoflagellates that were initially believed to be solely autotrophic have since been revealed as mixotrophs in the last decades [4, 6, 7]. The eco-physiological complexities of dinoflagellates make it difficult to understand their distributions, even though numerous dinoflagellate species regularly cause harmful algal blooms in coastal and offshore waters [8–10].

With the development of various observational technologies and sophisticated oceanographic surveys, it has been realized that dinoflagellates are an important group within the protistan communities in oligotrophic oceanic waters [11, 12]. High-throughput images of planktonic cells acquired by automated systems (e.g. Imaging FlowCytobot–ICFB, FlowCam, ZooScan) suggest the presence of large populations of dinoflagellates in offshore pelagic waters [13, 14]. Although the ribosomal DNA quantification of dinoflagellates is complicated due to higher gene copies than in other protists, 18S rRNA gene sequencing data also support a large presence of dinoflagellates along with high alpha-diversity in these regions [11, 15, 16]. However, considering their generally high iron requirements compared to other phytoplankton taxa [17, 18], this raises questions about the cellular mechanisms dinoflagellates use to survive in low-iron waters.

Globally, >20% of the open ocean belongs to high-nutrient, low-chlorophyll (HNLC) regions, where primary productivity is reduced compared to other areas that contain high macronutrient concentrations [19, 20] due to a scarcity of the micronutrient iron [19, 21]. Iron has also been identified as a key factor regulating phytoplankton growth in many coastal systems [22–24]. The California upwelling zone is a well-recognized seasonally iron-limited coastal system, largely dependent on upwelling dynamics [22, 25]. Thus, different phytoplankton taxa have independently adapted survival strategies in low-iron environments. One such strategy is the substantial reduction in the cellular iron requirements through substitution of iron-containing proteins for non-iron equivalents when cells are iron deficient [18, 26]. Picoeukaryotes can primarily benefit from their small size because it provides greater cellular surface area to volume ratios and higher nutrient uptake efficiencies [27]. Cyanobacteria can scavenge iron through the activation of siderophore-mediated high-affinity transport systems [28, 29], and many eukaryotic phytoplankton species can acquire iron from bacterial–algal mutualisms [30, 31]. Also, some diatoms contain the protein ferritin that provides for an extensive iron storage capacity [32].

To reveal potential strategies for utilizing iron of an ecologically significant dinoflagellate group, we examined members of one of the most common and cosmopolitan dinoflagellate genera, *Karlodinium*. Many *Karlodinium* species have been revealed to have mixotrophic capabilities, relying on both photosynthesis and phagotrophy for growth [6, 33, 34]. They are also notorious for causing fish-killing harmful algal blooms through their production of karlotoxin [34, 35]. Most importantly, *Karlodinium* are common species found in both coastal and offshore HNLC waters of the Northeast Pacific Ocean and elsewhere, thereby being an excellent representative for examining the response of dinoflagellates to iron deficiency [33, 35].

## Materials and methods

### Culture collection and conditions

A total of six isolates of the dinoflagellate species belonging to the genus *Karlodinium* were examined in this study (Table 1). Of the three oceanic isolates, two strains of *Karlodinium ballantinum* (UNC1835 & UNC1840) were isolated from HNLC water samples from Ocean Station Papa in the Northeast Pacific Ocean. The other oceanic isolate, *K. veneficum* (RCC6139), was isolated from waters in the Southwest Pacific Ocean, obtained from the Roscoff Culture Collection, France. Three coastal strains of *K. veneficum* isolated from the US coastal regions were also examined; both CCMP1975, which was isolated from the fish-killed waters of Fish Farm in Maryland, and CCMP2778, which was isolated from the coast of Longboat Key, were obtained from the NCMA at Bigelow Laboratory. The D3 strain was isolated from the Sweetwater River Estuary in San Diego, provided by the Place Laboratory at the University of Maryland.

**Table 1:** List of *Karlodinium* isolates with strain designations, sampling locations and dates, and 18S rDNA GenBank accession number.

*Karlodinium* spp.	Strain	Sampling location and date	Sampling date	Isolated temperature (°C)	Accession number
Oceanic					
*K. ballantinum*	UNC1835	Ocean Station Papa, Northeast Pacific Ocean (50° N, 145° W)	Sep 2018	14.0	PV240840
*K. ballantinum*	UNC1840	Ocean Station Papa, Northeast Pacific Ocean (50° N, 145° W)	Sep 2018	14.0	PV240841
*K. veneficum*	RCC6139	Chatham Rise, South Pacific Ocean (50 m depth, 45°31 S, 179°38 E)	Nov 2018	10.9	PV240842
Coastal					
*K. veneficum*	CCMP1975	Hyrock Farm near Manokin River, MD (38°10 N, 75°44 W)	Jul 1996	NA	FJ823568
*K. veneficum*	CCMP2778	Off Longboat Key near Sarasota, FL (27°19 N, 82°36 W)	Feb 2005	NA	EU165294
*K. veneficum*	D3	The mouth of the Sweetwater River, San Diego (32°38 N, 117°06 W)	Nov 2014	22.0	PV240843

NA, not available.

All *Karlodinium* cultures were grown in the synthetic seawater medium Aquil* using trace metal clean (TMC) techniques [36]. Vitamins and macronutrients were added to the medium following the standard Aquil* recipe [37]. Trace metals were buffered using 100 μmol l^−1^ of ethylenediaminetetraacetic acid (EDTA). Iron-replete treatments (+Fe) were prepared by adding 1370 nmol l^−1^ of total iron (FeT) in a 1:1 Fe: EDTA solution to Aquil medium, corresponding to a dissolved inorganic iron (Fe′) concentration of 2.7 nmol l^−1^. Iron-limited treatments (-Fe) were prepared by adding 12.9 nmol l^−1^ FeT, corresponding to a Fe′ concentration of 26 pmol l^−1^ [32].

Prior to experimentation, cultures were grown in acid-washed, Milli-Q water-rinsed 28 ml polycarbonate (PC) centrifuge tubes (Nalgene) and maintained in exponential phase by serial transfer. Irradiance was kept constant ~120μmol photons m^−2^ s^−1^ on a 14:10-h light:dark cycle. Cells were first acclimated to the iron concentrations (at least 10 transfers = 30–50 generations) at 12°C for oceanic strains and 20°C for coastal strains. Coastal and oceanic isolates were subsequently acclimated to 12°C and 20°C, respectively. Following this period, cultures were fully acclimated to experimental iron and temperature conditions for at least 1 month prior to experimentation.

Growth parameters in response to iron status and temperature were determined through measuring specific growth rates, maximum photochemical quantum efficiencies of photosystem II (*F_v_*/*F_m_*), and cell volumes. Intracellular iron contents were determined using an ^55^Fe label, radiotracer technique [38]. To assess mixotrophic ability, experiments were designed to investigate the potential grazing rates of oceanic and coastal *Karlodinium* species. Experiment 1 was designed to measure the specific growth rate of each *Karlodinium* strain under potentially mixotrophic and autotrophic conditions. Experiment 2 was designed to verify whether *Karlodinium* isolates actually fed on target prey species, using the remaining cultures from experiment 1 (containing mixtures of dinoflagellates and prey) incubated for 2 days. For detailed methods of each measurement, see supplementary information (Text S1).

### Gene expression analysis

Oceanic (UNC1840) and coastal (CCMP1975) strains of *Karlodinium* cells grown in acid-washed, Milli-Q water rinsed 2-l PC bottles (~1 × 10^7^ cells) were harvested for RNA analysis from four different iron and temperature combination treatments. In their exponential phase, cells were directly collected onto PC filters (Millipore; 0.45 μm pore size, 47 mm) using vacuum filtration and immediately flash frozen with liquid nitrogen and stored at −80°C until RNA extraction. Total RNA from each treatment (triplicate samples) was independently extracted using the RNAqueous 4-PCR Kit (Ambion) following the manufacturer’s protocols. Initial bead beating was conducted to detach cells from the filters and lyse cells. Residual genomic DNA was removed by incubating with DNase I at 37°C for 45 min and purified with DNase inactivation reagent (Ambion). Sample concentrations and RNA integrity numbers (RINs) were measured using an Agilent Bioanalyzer 2100, with RIN values exceeding 6.5. The mRNA libraries were constructed from ~2 μg of total RNA through poly-A selection and prepared using the TruSeq mRNA Library Preparation Kit (Illumina). Each sample was individually barcoded and pooled prior to paired-end RNA sequencing (2 × 150 bp) on a HiSeq 2000 System (Illumina) at the Genewiz Sequencing Facility (S. Plainfield, NJ). Prior to transcriptome assembly, raw reads were screened to remove low-quality read sequences using the Trimmomatic program [39].

The trimmed reads from the 12 libraries for each oceanic and coastal strain were *de novo* assembled with Trinity software [40]. Trinity assembled reads by merging overlapping sequences to generate longer, gap-free fragments, called contigs. Contigs with a minimum length of 201 bp were selected, and the longest among them were clustered into nonredundant transcripts using CD-HIT-EST, applying a clustering identity threshold of 0.98 to remove redundancy [41].

For gene annotation, sequence-based alignments were conducted using the BLASTx algorithm of the DIAMOND program [42], applying default settings and a cut-off *E* value of 1.00E-5 against the NCBI nonredundant (NCBI nr) database. Functional annotation of genes was further performed using the Gene Ontology and the Kyoto Encyclopedia of Genes and Genomes (KEGG) databases. Differential gene expression analysis was performed by calculating normalized counts, fold change values, and associated Benjamini–Hochberg adjusted *P*-values using DESeq2 [43]. Read counts were normalized within either the oceanic (UNC1840) or the coastal (CCMP1975) *Karlodinium* isolates using the “median ratio method” [43]. Pairwise comparisons were then made by measuring the Log (base 2) fold change of transcripts between Fe-replete and Fe-limited treatments. The Log_2_ fold change (adjusted *P*-value <.05) of specific genes of interest, particularly those involved in photosynthesis, nitrogen assimilation, and iron homeostasis were investigated in further detail at both the KEGG Ortholog (KO) and gene class level, with expression values averaged for multiple contigs sharing the same KO in RStudio (v.4.2.2).

## Results

### Growth rates of oceanic and coastal isolates

**Figure 1:**
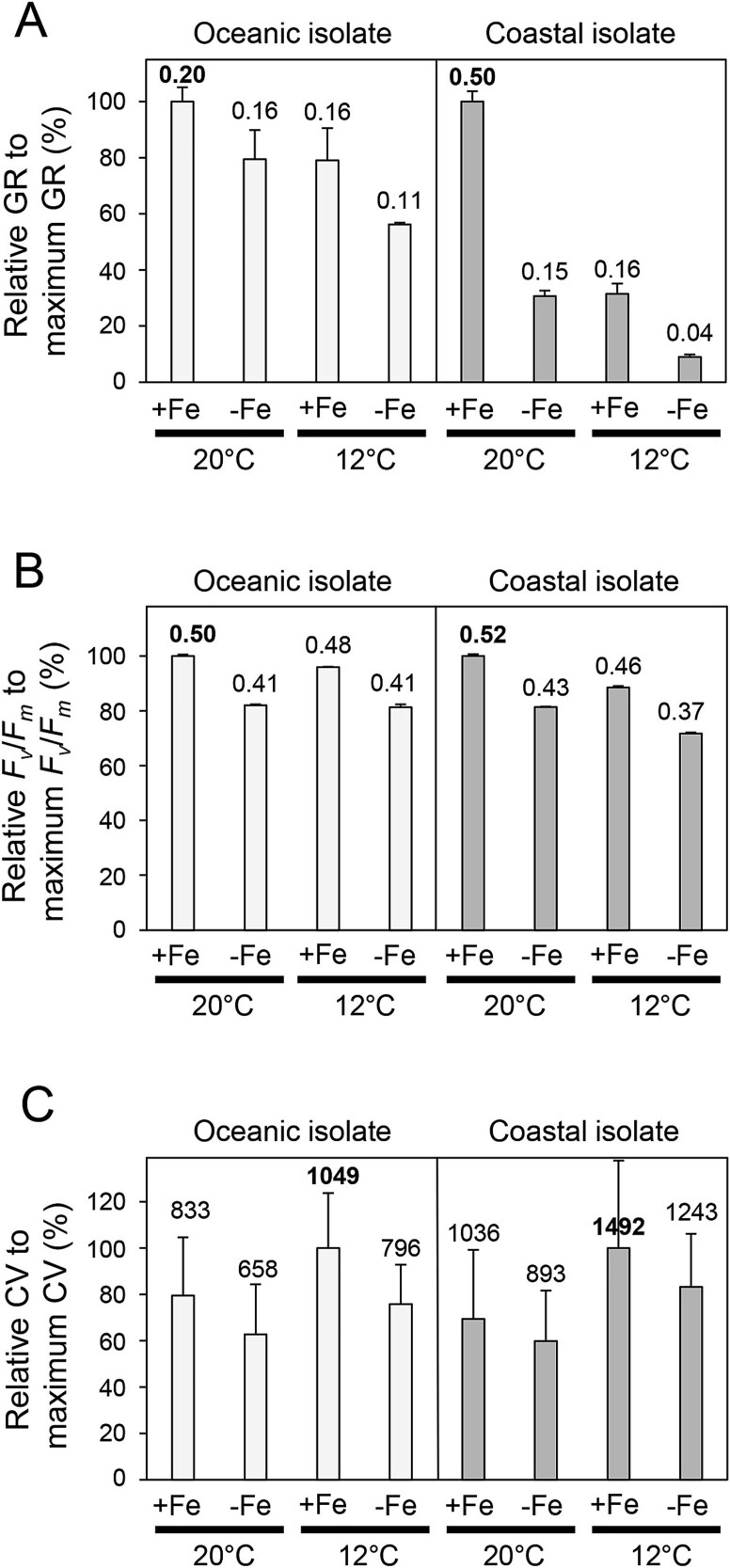
Growth parameters of representative oceanic (UNC1840) and coastal (CCMP1975) *Karlodinium* species. (A) Relative specific growth rates (GRs) measured based on microscopic cell counts, (B) relative maximum photochemical yield of PSII (*F*_v_/*F*_m_), and (C) relative cell volume (CV). The values above bars of (A), (B), and (C) are the absolute specific growth rate (μ, d^−1^), maximum photochemical quantum yield, and cell volume (μm^3^) in each treatment, respectively. The *y*-axis represents the relative values (%) compared to the maximum values under four different treatment conditions for each strain. Treatments are Fe-replete, 20°C (+Fe, 20°C), Fe-limited, 20°C (−Fe, 20°C), Fe-replete, 12°C (+Fe, 12°C), and Fe-limited, 12°C (−Fe, 12°C). Error bars indicate the standard deviation of biological replicates.

The growth rates of oceanic (UNC1840) and coastal (CCMP1975) isolates in response to iron availability and temperature were assessed by changes in cell density (Fig. 1A). The maximum specific growth rate (μ_max_) of the oceanic isolate was 0.20 d^−1^ under iron-replete (pFe 19) and warm-temperature (20°C) conditions. Under iron-limited (pFe 21) and cold temperature (12°C) conditions, its relative growth (μ/μ_max_) rates decreased to 55%–80% of μ_max_. In contrast, the coastal isolate had a higher μ_max_ of 0.50 d^−1^, but μ/μ_max_ declined more appreciably, ranging from 8% to 32% under iron-limiting conditions (pFe 21).

A similar trend was observed in additional measurements of growth rates using *in vivo* fluorescence for other oceanic and coastal strains (Supplementary Fig. S1). For oceanic strains, μ/μ_max_ in iron-limited and cold conditions ranged from 47.8% to 60.0%. In contrast, coastal strains exhibited more pronounced decreases, with the μ/μ_max_ of CCMP1975 dropping to 11.6%, matching the result observed from cell densities. Moreover, no growth was observed for CCMP2778 and D3 strains under low-iron conditions.

Under iron-replete conditions, low temperature (12°C) alone led to reduced growth rates across all six *Karlodinium* strains (Fig. 1A, Supplementary Fig. S1). Whereas oceanic isolates displayed only modest declines compared to their warm-temperature (20°C) growth rates, coastal isolates exhibited markedly larger reductions.

### Photosynthetic efficiency and cell volume

The photosynthetic efficiencies and cell volumes of the oceanic (UNC1840) and coastal isolate (CCMP1975) were specifically compared as a function of iron status and temperature conditions (Fig. 1B and C). The *F*_v_/*F*_m_ values of both oceanic and coastal isolates were highest in iron-replete and warmer conditions and lowest in iron-limited and colder conditions (Fig. 1B). However, there was no significant difference in the relative decrease in *F*_v_/*F*_m_ percentages between oceanic and coastal isolates under the different treatments.

Cell volume of the oceanic isolate was slightly smaller than the coastal isolate, both of which were largest in the iron-replete and colder treatment (Fig. 1C). No significant difference was observed in the relative decrease in cell volume percentages between oceanic and coastal isolates under the different iron and temperature conditions.

### Intracellular elemental quotas and stoichiometry

**Figure 2:**
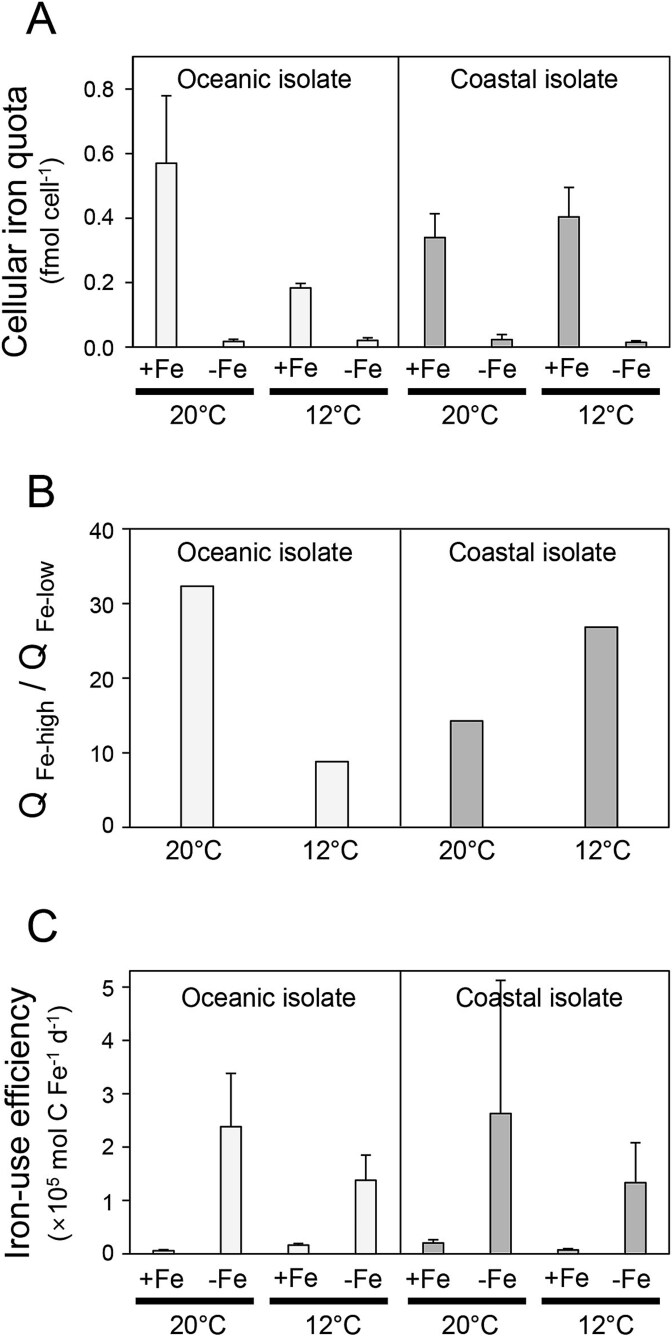
Iron content parameters of representative oceanic (UNC1840) and coastal (CCMP1975) *Karlodinium* species. (A) Intracellular iron content (fmol cell^−1^), (B) iron quota ratios under Fe-replete and Fe-limited conditions (Fe-Q_high_: Fe-Q_low_) at different temperatures, and (C) iron-use efficiency, defined as the rate of cellular carbon per unit of cellular iron per day (×10^5^ mol C Fe^−1^ d^−1^). Experimental conditions include Fe-replete, 20°C (+Fe, 20°C), Fe-limited, 20°C (−Fe, 20°C), Fe-replete, 12°C (+Fe, 12°C), and Fe-limited, 12°C (−Fe, 12°C). Error bars indicate the standard deviation of biological replicates.

The elemental contents (cellular Fe, C, and N) were expressed both per cell and per unit cell volume to account for variations in cell volume (Table 2). In iron-limited conditions, the cell quotas of Fe in both the oceanic and coastal isolates were significantly decreased regardless of temperature treatment (Table 2, Fig. 2A). Specifically, under iron-replete conditions, the Fe quotas for oceanic and coastal isolates were 0.18–0.57 and 0.34–0.40 fmol cell^−1^, respectively. However, under iron-limited growth conditions, the Fe quotas for both oceanic and coastal isolates significantly decreased to 0.015–0.024 fmol cell^−1^, representing only 3%–11% of the values observed under iron-replete conditions. Temperature differences did not show a clear trend in Fe cell quotas.

**Table 2:** Iron (Fe), carbon (C), and nitrogen (N) quotas of oceanic and coastal *Karlodinium* strains grown in different temperatures (*T*) and iron-replete (pFe 19) and iron-limited (pFe 21) conditions. Fe, C, and N quotas are normalized per cell and per unit cell volume. Quotas are presented as the averages ± standard deviations.

Strain	*T* (°C)	[Fe]_Total_ (nmol L^−1^)	pFe^*^	Cell volume (μm^−3^)	Fe		C		N	
					(fmol cell^−1^)	(amol μm^−3^)	(pmol cell^−1^)	(fmol μm^−3^)	(pmol cell^−1^)	(fmol μm^−3^)
Oceanic (UNC1840)										
	20	1370	19	833.4 ± 264.8	0.57 ± 0.21	0.68 ± 0.33	14.5 ± 1.3	17.36 ± 5.74	3.6 ± 0.5	4.29 ± 1.51
	20	12.9	21	658.3 ± 227.7	0.02 ± 0.01	0.02 ± 0.01	23.3 ± 2.7	35.38 ± 12.93	9.0 ± 1.7	13.72 ± 5.37
	12	1370	19	1049.0 ± 249.8	0.18 ± 0.01	0.22 ± 0.06	18.8 ± 3.1	17.93 ± 5.20	6.9 ± 2.6	6.58 ± 2.89
	12	12.9	21	796.0 ± 177.1	0.02 ± 0.01	0.02 ± 0.01	16.9 ± 0.7	21.18 ± 4.80	4.8 ± 1.2	5.99 ± 2.03
Coastal (CCMP1975)										
	20	1370	19	1036.2 ± 444.1	0.34 ± 0.07	0.41 ± 0.20	13.3 ± 2.5	12.82 ± 6.02	2.3 ± 0.5	2.22 ± 1.06
	20	12.9	21	893.1 ± 326.0	0.02 ± 0.02	0.03 ± 0.02	26.8 ± 18.1	30.03 ± 23.06	3.7 ± 2.2	4.17 ± 2.89
	12	1370	19	1492.2 ± 563.2	0.40 ± 0.09	0.48 ± 0.21	17.6 ± 5.2	11.78 ± 5.64	2.6 ± 0.8	1.72 ± 0.83
	12	12.9	21	1243.5 ± 341.3	0.02 ± 0.01	0.03 ± 0.01	55.0 ± 22.0	44.19 ± 20.20	7.0 ± 2.5	5.64 ± 2.54

Note. The standard errors of the averaged quotas normalized by unit cell volume were calculated through the propagation of error formula [73] due to each cellular quota (*n* > 3) and cell volume (*n* > 30) being measured by independent experiments.

The cellular quotas of C and N appeared to generally increase under iron-limited conditions, although most differences were not significant whereas quotas did not show a trend at the different temperatures (Table 2). Additionally, when examining the Fe quota ratios under Fe-replete and Fe-limited conditions (Fe-Q_high_: Fe-Q_low_) at each temperature, no significant difference was observed between the oceanic and the coastal isolates, with a higher ratio in the oceanic isolate at 20°C and in the coastal isolate at 12°C. Iron quota ratios ranged from 8.8 to 32.3 in the oceanic and 14.3 to 26.8 in the coastal isolate in the different temperature treatments. (Fig. 2B).

In iron-replete conditions, the intracellular Fe:C and Fe:N ratios of oceanic and coastal isolates ranged from 9.8 to 39.4 μmol Fe mol C^−1^ and from 26.6 to 159.3 μmol Fe mol N^−1^, respectively, showing no significant differences (Table 3). However, under low-iron conditions, both Fe:C and Fe:N ratios of oceanic and coastal isolates decreased appreciably compared to those of iron-replete cells. The Fe:C and Fe:N ratios were only 2%–9% and 1%–12% of those measured in high-iron conditions, respectively. The C:N ratios of both oceanic and coastal isolates were not significantly affected by the availability of iron and temperature conditions. Nevertheless, the coastal isolate exhibited C:N ratios ranging from 5.8 to 7.8 (6.9 ± 0.6) mol C mol N^−1^, consistent with the Redfield ratio, whereas the oceanic isolate displayed markedly lower ratios ranging from 2.6 to 4.1 (3.3 ± 0.4) mol C mol N^−1^.

**Table 3:** Mean specific growth rates, iron (Fe): carbon (C), Fe: nitrogen (N), and C: N ratios, and iron-use efficiencies of examined oceanic and coastal *Karlodinium* strains grown in different temperatures (*T*) and iron-replete (pFe 19) and iron-limited (pFe 21) conditions.

Strain	*T* (°C)	[Fe]_Total_ (nmol L^−1^)	pFe^a^	Growth rate (d^−1^)	Fe: C ratio (μmol Fe mol C^−1^)	Fe: N ratio (μmol Fe mol N^−1^)	C: N ratio (mol C mol N^−1^)	Iron-use efficiency^b^ (×10^5^ mol C Fe^−1^ d^−1^)
Oceanic (UNC1840)								
	20	1370	19	0.20 ± 0.01	39.4 ± 14.9	159.3 ± 63.0	4.1 ± 0.3	0.06 ± 0.02
	20	12.9	21	0.16 ± 0.02	0.8 ± 0.3	2.0 ± 0.9	2.6 ± 0.2	2.38 ± 1.00
	12	1370	19	0.16 ± 0.02	9.8 ± 1.8	26.6 ± 10.0	2.9 ± 0.6	0.16 ± 0.03
	12	12.9	21	0.11 ± 0.00	0.9 ± 0.3	3.2 ± 1.3	3.7 ± 1.0	1.38 ± 0.47
Coastal (CCMP1975)								
	20	1370	19	0.50 ± 0.02	25.6 ± 7.4	147.9 ± 44.5	5.8 ± 0.3	0.20 ± 0.06
	20	12.9	21	0.15 ± 0.01	0.9 ± 0.8	6.4 ± 5.7	6.9 ± 0.7	2.63 ± 2.50
	12	1370	19	0.16 ± 0.02	23.0 ± 8.5	157.5 ± 58.7	6.9 ± 0.01	0.07 ± 0.03
	12	12.9	21	0.04 ± 0.01	0.4 ± 0.2	3.0 ± 1.6	7.8 ± 1.5	1.34 ± 0.74

a pFe = −Log[Fe^3+^].

b The mean iron-use efficiencies shown are the averages of independently calculated values derived from the *n* Fe: C ratios and their corresponding growth rates.

Iron-use efficiency (IUE) was expressed as the rate of carbon assimilated per unit of cellular iron per day and is derived from the cellular C and Fe quotas and growth rates (Table 3, Fig. 2C). The IUEs of both oceanic and coastal isolates were appreciably higher in the iron-limited treatments with values slightly higher at the warmer temperature. There was no statistical difference in the IUEs between oceanic and coastal isolates under different iron and temperature conditions.

### Mixotrophic abilities of oceanic and coastal isolates

To investigate the potential particulate iron acquisition ability of *Karlodinium* species through mixotrophy, we compared mixotrophic growth rates and percentages of the prey-material ingested cells among six isolates (Table 4). Among coastal strains, mixotrophic growth rates of the two isolates (CCMP1975 and D3) when fed on two prey species, *Rhodomonas salina* and/or *Heterosigma akashiwo*, were significantly higher than each autotrophic growth rate (Student's *t*-test, *P* < .05). Although the mixotrophic growth rate of another coastal isolate (CCMP2778) was not significantly different from its autotrophic growth rate, the inoculated algal prey materials were observed in the protoplasm of all three coastal isolates (Supplementary Fig. S2). In contrast, the growth rates of the three oceanic isolates when potential prey species were inoculated did not show a statistical difference compared to their autotrophic growth rate. Although UNC1835 and UNC1840 oceanic strains showed a tendency to attack various inoculated prey species (e.g. *R. salina, H. akashiwo, Alexandrium fundyense*, and *Pseudo-nitzschia subcurvata*), no evidence of prey materials was found within their protoplasms as measured under Fe-replete conditions (Table 4) or qualitatively observed under Fe-limiting conditions (data not shown). Similarly, the RCC6139 oceanic strain exhibited no evidence of mixotrophic ability, as no cells containing prey material observed, and its swimming behavior remained unaltered upon encountering inoculated microalgal species.

**Table 4:** List of *Karlodinium* strains with their mixotrophic tendencies (i.e. proportions of cells containing food vacuoles and mixotrophic growth rates) and autotrophic growth rates. The experiments were conducted under temperature conditions similar to those in the isolated marine environments (i.e. oceanic isolates, 12°C; coastal isolates, 20°C) and iron-replete conditions.

*Karlodinium* spp.	Strain	Proportions of cells containing food vacuoles (%)		Potential mixotrophic growth rates		Autotrophic growth rates
		*R. salina*	*H. akashiwo*	*R. salina*	*H. akashiwo*	
Oceanic						
*K. ballantinum*	UNC1835	0	0	0.21 ± 0.05	0.23 ± 0.01	0.21 ± 0.03
*K. ballantinum*	UNC1840	0	0	0.21 ± 0.06	0.21 ± 0.05	0.19 ± 0.02
*K. veneficum*	RCC6139	0	0	0.08 ± 0.02	0.05 ± 0.04	0.09 ± 0.02
Coastal						
*K. veneficum*	CCMP1975	35	37	0.46 ± 0.01	0.52 ± 0.01	0.34 ± 0.02
*K. veneficum*	D3	47	29	0.37 ± 0.01		0.24 ± 0.02
*K. veneficum*	CCMP2778	16	12	0.18 ± 0.03		0.15 ± 0.07

### Differential gene expressions

**Figure 3:**
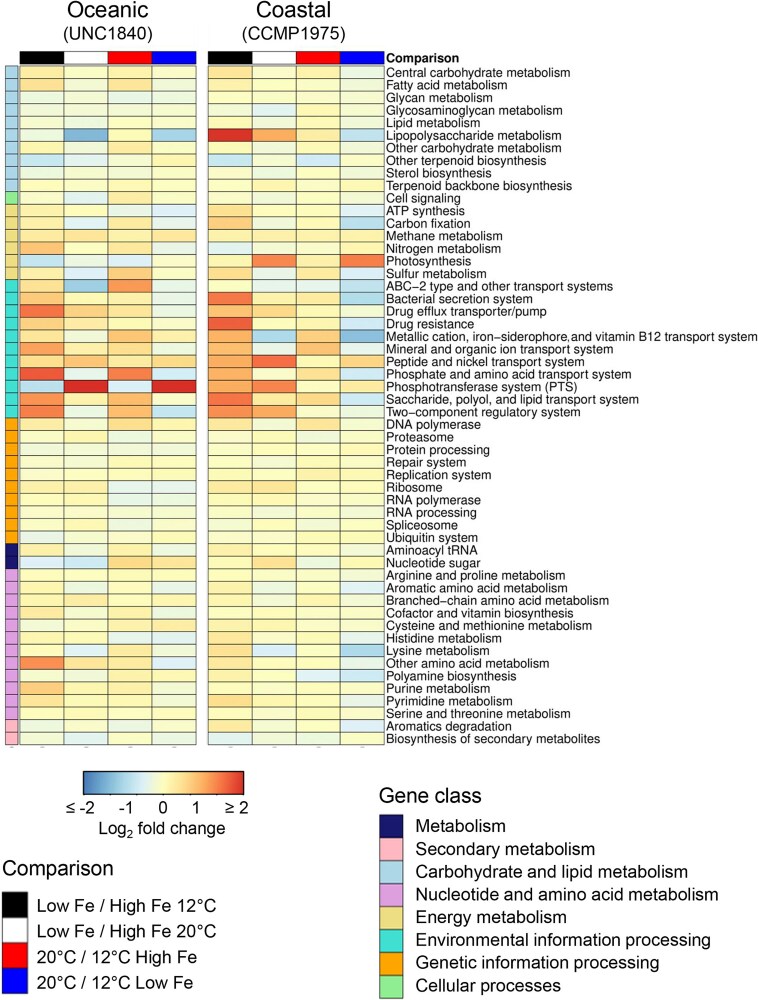
Differential expression of class II (gene class) and class III level pathways (assigned by KEGG modules) between the UNC1840 (oceanic) and CCMP1975 (coastal) *Karlodinium* isolates. Columns denote the fold change of transcripts in iron-limited (low-Fe) versus iron-replete (high Fe) treatments at 12°C and 20°C. The heatmap indicates the Log_2_ fold change in gene expression of combined genes belonging to each pathway. Pathways are categorized into gene classes according to KEGG.

To investigate the differential gene expression responses between the oceanic (UNC1840) and coastal (CCMP1975) isolates of *Karlodinium*, a KEGG pathway analysis was conducted (Fig. 3). Both strains demonstrated substantial variability in the expression of pathways associated with “environmental information processing,” indicating the central role of transport and signaling systems in response to low iron conditions. The “lipopolysaccharide metabolism” pathway was also uniquely over-represented in the coastal isolate under iron-limited growth conditions. In contrast, in the oceanic isolate, genes in the “other amino acid metabolism” pathway were over-represented under iron-limited conditions, alongside decreased expression of genes belonging to the “nucleotide sugar” pathway.

**Figure 4:**
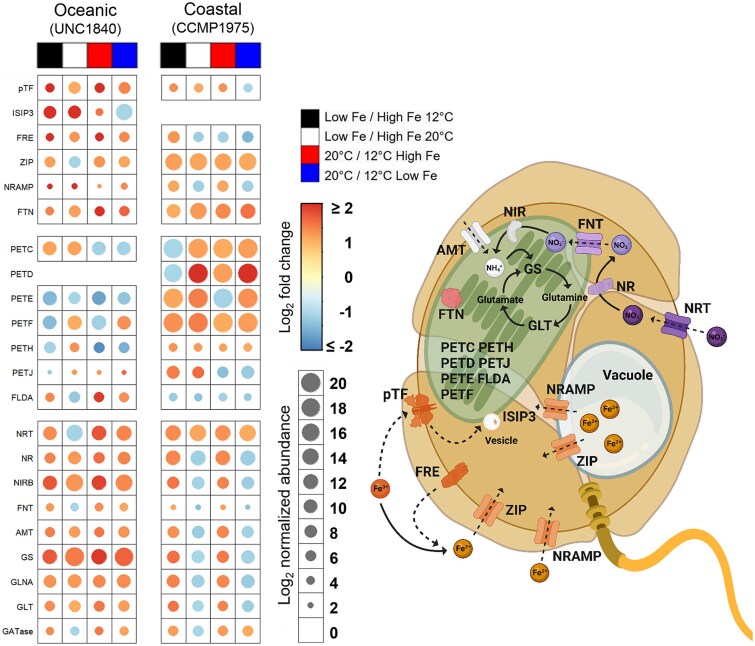
Overview of transcriptional patterns and predicted subcellular localization of key proteins in oceanic (UNC1840) and coastal (CCMP1975) *Karlodinium* isolates under different iron and temperature conditions. Bubble heatmaps of the oceanic and coastal isolates depicting gene expression (Log_2_ fold change and Log_2_ normalized abundance) for genes involved in iron homeostasis, nitrogen assimilation, and photosynthesis. Columns denote the fold change of transcripts in iron-limited (low Fe) versus iron-replete (high Fe) incubations at 12°C and 20°C. The heatmap indicates the Log_2_ fold change in gene expression, and the size of the circles indicates the Log_2_ normalized transcript abundance. On the right is a cellular schematic of the putative localizations of the different proteins listed for iron homeostasis (orange proteins), nitrogen assimilation (purple proteins), and photosynthesis found in *Karlodinium* dinoflagellates. Created with BioRender.com. Gene/protein name for each abbreviation is provided in Supplementary Table S1.

Bubble heatmaps illustrated the responses of specific genes related to iron homeostasis, photosynthesis, and nitrogen assimilation to low-iron conditions and temperature variations, elucidating gene expression patterns (Log_2_ fold change) and transcript abundances (Log_2_ normalized abundance) (Fig. 4). Overall, the coastal isolate exhibited higher normalized transcript abundances, particularly for photosynthetic genes, although the fold changes in expression were less pronounced. In contrast, the oceanic isolate demonstrated lower normalized transcript abundances of photosynthetic genes but displayed greater fold changes in gene expression.

In relation to genes involved in iron acquisition, the iron-starved induced protein 3 (*ISIP3*) was only detected and expressed in the oceanic isolate and displayed significantly increased expression under low iron conditions (Fig. 4). Furthermore, genes associated with iron acquisition and transport displayed an overall increase pattern under low iron in both oceanic and coastal isolates. Specifically, the oceanic isolate more routinely increased gene expression of numerous protein-encoding genes involved in iron acquisition (e.g. *pTF*, *ISIP3*, *FRE*, and *NRAMP*), whereas both isolates exhibited higher transcript abundance of an iron transporter protein (*ZIP*) and ferritin (*FTN*), an iron storage protein.

In response to iron limitation, the transcriptional profiles of photosynthesis-related genes displayed marked differences between the oceanic and coastal isolates. In the oceanic isolate, cytochrome b6f (*PETC*) and flavodoxin (*FLDA*) increased in gene expression, whereas cytochrome b6-f complex subunit 4 (*PETD*) was not detected under these culture conditions. Additionally, other genes encoding photosynthetic proteins (e.g. *PETE*, *PETF*, and *PETJ*) displayed reduced gene expression levels. In contrast, the coastal isolate demonstrated consistently high transcript abundance for photosynthesis-related genes such as *PETC*, *PETD*, plastocyanin (*PETE)*, and ferredoxin (*PETF*), irrespective of iron availability or temperature changes. Under iron-limited conditions, the expression levels of *PETE*, *PETF*, *PETH*, and cytochrome c6 (*PETJ*) further increased, whereas *FLDA* expression decreased.

The gene expression responses of nitrogen metabolism-related genes under varying iron and temperature conditions also revealed distinct patterns between the oceanic and coastal isolates. In the oceanic isolate, the majority of genes, including nitrate reductase (*NR*), nitrite reductase (*NIRB*), ammonium transporter (*AMT*), glutamine synthetase (*GS*), the GS-encoding gene (*GLNA*), and *GLT* (glutamate transporter), exhibited increased gene expression under low iron conditions. Among them, *NIRB*, *GS*, and *GLNA* exhibited not only increased expression levels but also higher transcript abundances, indicating a broad nitrogen assimilation response. A distinct observation was that elevated temperature (20°C) for the oceanic isolate also resulted in increased expression of most nitrogen metabolism genes compared to 12°C, regardless of iron availability. In contrast, the coastal isolate demonstrated substantial over-representation of nitrogen metabolism-related genes specifically under the condition of 20°C under high iron conditions and when compared to 12°C, reflecting its optimal growth conditions. Under low iron conditions at 12°C, nitrogen-related genes were also over-represented, suggesting a compensatory response to environmental stress.

## Discussion

Based on the results of this study, the oceanic isolates exhibited higher relative growth rates (i.e. μ/μ_max_) compared to their coastal counterparts when subjected to conditions of low iron availability, implying that the oceanic isolates possess an enhanced capacity to better cope under situations where iron is low and limiting to growth. Thus, to elucidate potential mechanisms that these isolates utilize, we conducted a thorough analysis of their physiological and molecular attributes, alongside comparisons with other similar protistan groups.

### Enhanced growth rates of oceanic isolates under iron-limited conditions

The distinct growth patterns observed between oceanic and coastal isolates under varying iron and temperature conditions highlight their divergent ecological adaptations. Although coastal isolates grew faster, oceanic isolates demonstrated superior relative growth rates under iron-deficient and cold conditions, where μ/μ_max_ was much higher, suggesting their adaptation to subarctic/subantarctic iron-limited waters. This observation is consistent with the frequent dominance of larger dinoflagellate taxa in these regions [44–46]. Conversely, coastal isolates, despite achieving higher growth rates under optimal conditions, showed markedly reduced or entirely inhibited growth under iron-deficient conditions, highlighting their comparably limited ability to modify cellular iron requirements or uptake strategies, perhaps as a consequence of the elevated iron levels typically found in coastal environments [18].

Although our study primarily focused on iron availability, the reduced growth rates at low temperature (12°C), even under iron-replete conditions, suggests that suboptimal temperature may act as a colimiting factor. *Karlodinium* species typically grow best at 20°C–25°C [47, 48], and the sharper decline in the coastal strains at 12°C, compared to the oceanic strains, indicates greater thermal sensitivity. This divergence implies that temperature may modulate physiological responses to iron stress, potentially through impacts on nutrient uptake or cellular metabolism. Indeed, such interactive effects (i.e. low temperature exacerbates iron limitation) have been reported in other protists [49–51]. As dinoflagellates often perform better under warmer conditions [52, 53], continued ocean warming may help mitigate iron stress in certain species, although such interactive responses remain to be confirmed.

Despite limited comparative studies on growth characteristics between oceanic and coastal isolates within dinoflagellates, research on diatoms has revealed that oceanic isolates exhibit enhanced adaptability to iron scarcity, reflecting the selective pressures of their respective habitats [36, 54]. In general, across various protistan taxa, oceanic isolates appear to have developed adaptations to cope with environmental stressors, whereas coastal isolates are optimized for rapid growth in iron-rich conditions, likely benefiting due to the sufficient iron availability.

### Physiological responses to iron deficiency

Contrary to the expectation that the oceanic strain would exhibit superior physiological adaptations to low-iron conditions, our findings indicate that both oceanic and coastal *Karlodinium* strains employ similar physiological mechanisms to cope with iron limitation. Both strains exhibited reduced photosynthetic efficiency (*F*_v_/*F*_m_) and cell volume under iron limitation, reflecting common stress responses likely associated with impaired photosystem function and cellular adjustments to enhance nutrient uptake efficiency [27, 55]. Although such responses are consistent with adaptations observed in other protist groups [26, 55, 56], the absence of a distinct advantage in the oceanic strain suggests that physiological traits alone may not fully explain its adaptation to persistently iron-limited environments.

The moderate decline in *F*_v_*/F*_m_ may reflect a dinoflagellate-specific resilience. Certain dinoflagellates maintain relatively stable *F*_v_*/F*_m_ under nutrient stress compared to other protistan groups [57]. Similarly, *A. catenella* and *K. veneficum* exhibit only modest declines under iron and phosphorus limitation, respectively, likely due to photoprotective mechanisms such as iron chelate utilization and enhanced nonphotochemical quenching [58, 59]. Additionally, the extent of decline in *F*_v_*/F*_m_ relative to growth rate reduction can vary among iron-limited phytoplankton [36].

We also observed low C:N ratios in the oceanic isolate. Although these values may appear anomalous, previous studies have reported similarly low values in dinoflagellates—below the Redfield ratio of 6.6—with some species ranging from 3.4 to 6.5 [60]. *Karlodinium* species in particular exhibit a range of 4.0–7.0, with a median of ~4.75 [61]. These findings suggest that the observed values fall within the expected range for this group, potentially reflecting taxon-specific stoichiometry or enhanced N storage. Such physiological flexibility may contribute to the persistence of oceanic isolates in oligotrophic, iron-limited environments and warrants further investigation.

Both strains demonstrated significantly increased iron-use efficiencies under low-iron conditions, which may reflect a generalized compensatory response to mitigate iron stress rather than a specific optimization. This suggests that even though oceanic strains are presumed to have evolved under chronic iron limitation, the coastal strains’ comparable IUE increase highlights its inherent flexibility to handle episodic iron scarcity. This finding contrasts with other oceanic protists, such as diatoms, which often exhibit enhanced IUE in nutrient-poor environments [36, 54]. Although the oceanic strain’s molecular-level adaptations may provide advantages in iron acquisition and regulation (see gene expression analysis below), these do not manifest as measurable physiological differences under the conditions tested. Instead, both strains effectively balance reductions in photosynthetic efficiency, cell volume, and intracellular iron content with increased IUE to survive iron-limited conditions.

### Mixotrophic abilities of oceanic and coastal isolates

Mixotrophy is often considered an adaptive strategy that allows phytoplankton to supplement photosynthesis with phagotrophy, potentially providing an advantage under nutrient limitation, including iron scarcity [62, 63]. However, our results reveal a contrast between oceanic and coastal *Karlodinium* isolates. Whereas all three coastal strains demonstrated clear mixotrophic growth, oceanic strains exhibited no evidence of phagotrophy, despite interactions with potential prey. This suggests that mixotrophy is an important trait for some coastal *Karlodinium* strains but is not a universal feature of the genus.

The absence of mixotrophy in oceanic strains suggests an alternative iron acquisition strategy. Given that particulate iron is a significant component of bioavailable iron in marine environments [63, 64], phagotrophy could have provided an advantage. However, oceanic *Karlodinium* isolates may rely entirely on dissolved iron uptake, potentially through highly efficient iron transport systems. Their long-term adaptation to oligotrophic waters, where particulate organic matter is scarce, may have made the energetic cost of maintaining mixotrophy impractical, ultimately leading to the loss of their phagotrophic ability over time.

Despite differences in mixotrophic ability, both oceanic and coastal strains exhibited similar physiological responses to iron limitation, suggesting that although mixotrophy provides a clear advantage for coastal strains under nutrient-replete conditions, it may not be a crucial determinant of survival under iron scarcity. Instead, coastal strains may utilize mixotrophy opportunistically in nutrient-rich conditions, whereas oceanic strains have evolved alternative mechanisms for iron acquisition. This divergence underscores the niche-specific nature of mixotrophy, reinforcing that it is not a universally beneficial trait but rather an adaptation to dynamic coastal environments.

### Transcriptional responses to iron deficiency

Metabolic pathway analysis as examined through gene expression revealed that under low iron and variable temperature conditions, pathways associated with “environmental information processing” appear to be fundamentally critical for sensing external stresses, acquiring resources such as nutrients, and modulating cellular processes to ensure survival, irrespective of geographically distinct adaptive strains. Supporting these findings, several studies have demonstrated that pathways within this category, including signal transduction systems (e.g. two-component regulatory system) for sensing nutrient depletion and transporter systems (e.g. ABC transporters and phosphotransferase system) for enhancing the uptake of scarce nutrients, are activated across various protist species under nutrient-limited conditions [65, 66].

Further insights into ecological adaptation strategies to iron limitation were revealed through the transcriptional regulation patterns of sentinel genes. The coastal isolate, which commonly thrives in relatively iron-replete conditions, exhibited overall high normalized gene transcript abundances, suggesting a robust mechanism for maintaining metabolic activity under iron-limiting conditions. This adaptation likely facilitates rapid recovery and restoration of metabolic functions in environments with fluctuating iron levels. Conversely, the oceanic isolate, adapted to persistently iron-scarce environments, displayed lower normalized transcript abundances but greater fold changes in gene expression. This pattern suggests a resource-efficient strategy, reflecting an adaptation to chronic iron limitation where transcriptional regulation is finely adjusted to conserve resources, thus sustaining survival in a consistently nutrient-limited environment.

The differential expression patterns of iron acquisition genes between the oceanic and coastal isolates reveal distinct adaptive strategies to iron limitation. Of note, *ISIP3*, putatively an iron storage and/or uptake protein not previously identified in dinoflagellates [67, 68], was exclusively identified and highly overrepresented in the oceanic isolate under iron-deficient conditions, suggesting a specialized adaptation to chronically low-iron environments. The increased expression of *pTF*, *FRE*, and *NRAMP* further supports a high-affinity iron uptake system in oceanic *Karlodinium*, suggesting it is optimized for efficient acquisition and intracellular transport of scarce iron resources, consistent with adaptation to HNLC regions. In contrast, in the coastal isolate, the overrepresentation of transcripts encoding *ZIP*, an iron transporter, and *FTN*, an iron storage protein, suggests mechanisms for the rapid acquisition and/or storage of iron. This strategy aligns with the coastal isolate’s adaptation to dynamic environments where iron levels vary due to upwelling or riverine inputs [22, 24, 69].

The transcriptional patterns of photosynthetic genes also reveal distinct adaptations to iron-deficient environments in the oceanic and coastal isolates. The oceanic *Karlodinium* displays increased expression of *FLDA* compared to the coastal *Karlodinium*, suggesting reliance on flavodoxin as an alternative to the iron-dependent equivalent ferredoxin (*PETF*). The absence of *PETD* and reduced expression of *PETF* and *PETJ* indicate a strategy to minimize iron-requiring proteins, optimizing survival in persistently iron-poor conditions. Conversely, the coastal strain demonstrates high transcript abundance of *PETC*, *PETD*, *PETE*, and *PETF*, likely trying to maintain photosynthetic activity under iron-limited conditions. Apart from a preference for plastocyanin, which can substitute for cytochrome c6 and contains copper instead of iron [70], increased fold changes of *PETF*, *PETH*, and *PETJ* suggest continued dependence on ferredoxin-related pathways, whereas reduced *FLDA* expression indicates an inability to reduce iron requirements. Overall, the oceanic strain adopts a resource-efficient approach, prioritizing more iron-independent proteins, whereas the coastal strain adapts to fluctuating iron levels by retaining diverse photosynthetic components for rapid recovery.

In relation to nitrogen metabolism, the oceanic isolate consistently expressed key nitrogen assimilation genes, such as *NIRB*, *GS*, and *GLNA*, particularly under iron-deficient conditions, highlighting an efficient nitrogen assimilation strategy suited to nutrient-scarce open-ocean ecosystems. This response was further amplified at elevated temperatures, demonstrating the strain’s resilience to simultaneous iron and temperature stress. In contrast, the coastal isolate exhibited a more conditional response, with significant overrepresentation of nitrogen assimilation genes only at optimal temperatures (20°C) under iron-sufficient conditions, reflecting its adaptation to dynamic and nutrient-rich coastal environments. Under iron-limited and lower temperature conditions (12°C), the coastal strain displayed a compensatory expression pattern, indicative of an adaptive response to stress, albeit less robust than that of the oceanic strain. Overall, the coastal strain’s temperature-dependent transcriptional regulation reflects a trade-off between metabolic efficiency and environmental adaptability, allowing it to exploit favorable conditions but limiting its performance under stressful ones, whereas the oceanic strain has the ability to uniformly highly express nitrogen assimilation genes under dual stress from low iron and elevated temperature, potentially underscoring its specialization in maintaining metabolic homeostasis.

## Conclusions

This study reveals distinct, yet overlapping iron acquisition strategies in oceanic and coastal *Karlodinium* isolates, emphasizing their unique ecological adaptations to iron limitation. Although oceanic isolates exhibited reduced susceptibility to iron limitation by maintaining relatively higher growth rates, their physiological responses were largely comparable to those of coastal strains, suggesting shared iron stress mitigation mechanisms. Both isolates effectively reduced cellular iron demands and enhanced iron-use efficiency, yet the lack of pronounced physiological advantages in oceanic strains suggests that their adaptation is driven primarily by molecular regulation. Transcriptomic analyses suggest that oceanic strains rely on a high-affinity iron uptake system for iron acquisition rather than mixotrophy, distinguishing their survival strategies from those of coastal counterparts. These findings highlight the complexity of iron homeostasis in mixotrophic dinoflagellates and provide new insights into their persistence in iron-deprived marine environments.

Although this study provides new insights into strain-level variation in iron acquisition and mixotrophy within *Karlodinium*, its conclusions are based on a limited number of isolates of a single genus. In natural communities, a variety of environmental factors such as prey diversity, variable light conditions, and colimitation by other nutrients may influence iron acquisition or mixotrophic behavior in ways not fully captured under controlled laboratory conditions. Moreover, dinoflagellates exhibit considerable diversity in nutritional strategies and iron requirements [16, 30, 71, 72]. Nonetheless, our results are consistent with broader patterns observed in other protist lineages, such as enhanced iron-use efficiencies in oceanic isolates. The observed divergence in mixotrophic capacity between strains may represent an additional ecological trade-off, though such patterns remain underexplored across taxa. Future comparative studies across multiple taxa and environmental gradients are essential to assess the ecological generality of these findings.

## Supplementary Material

Supplementary_information_Jang_et_al_2025_wraf099

## Data Availability

The datasets presented in this study are available in the NCBI online repository. The transcriptomic raw read data can be accessed under the BioProject accession numbers PRJNA1259046 for the oceanic strain and PRJNA1259047 for the coastal strain. The ribosomal DNA sequences are listed under the accession numbers provided in Table 1.
