# Supplementary material for: Distinct iron acquisition strategies in oceanic and coastal variants of the mixotrophic dinoflagellate Karlodinium
Source: ISME J. 2025 May 20;19(1):wraf099. doi: 10.1093/ismejo/wraf099 (PMC12161497; doi:10.1093/ismejo/wraf099)
Supplement: Supplementary_information_Jang_et_al_2025_wraf099 [file supplementary_information_jang_et_al_2025_wraf099.pdf]

Supplementary Information for

Distinct iron acquisition strategies in oceanic and coastal variants of the mixotrophic dinoflagellate *Karlodinium*

Se Hyeon Jang[1,2], YuanYu Lin[1], Adrian Marchetti[1]

[1]Department of Earth, Marine and Environmental Sciences, University of North Carolina at Chapel Hill, Chapel Hill, NC, 27514, USA

[2]Department of Oceanography, Chonnam National University, Gwangju 61186, Republic of Korea

***For correspondence:**

Dr. Adrian Marchetti

Department of Earth, Marine and Environmental Sciences

University of North Carolina at Chapel Hill

CB 3300

Chapel Hill, NC 27599-3300, USA

Email: amarchetti@unc.edu

Tel. (+1) 919 843 3473

Fax (+1) 919 962 1254

Supplementary Information

Supplementary Text S1

Growth, photosynthetic efficiency, and cell volume

To reduce possible iron contamination, during the culture maintenance period, growth rates were estimated by directly measuring *in vivo* relative chlorophyll-*a* fluorescence unit (RFUs) within enclosed 28 mL culture tubes using a 10-AU fluorometer [1, 2]. For the main experimentation, cultures were incubated using 2L acid-washed (1 mol L^{-1} HCl), Milli-Q water (Millipore)-rinsed PC bottles. Cell counts were carried out using an Olympus CKX-31 inverted light microscope. Because the growth rates of *Karlodinium* strains were generally cell density-dependent, initial inoculated cell concentrations were kept as similar as possible among experiments (approximately 2,000–3,000 cells mL^{-1}). Every 2 days, 3-mL aliquots were taken under a TMC laminar flow hood and fixed with Lugol's solution (final conc. 5%). The aliquots were taken from the fixed samples and then transferred to 1-mL Sedgwick-Rafter (SR) chambers. More than 300 cells on the SR chambers were enumerated to determine the abundance of cells. Specific growth rates (d^{-1}) were calculated from linear regression of the natural log cell density versus time during the exponential growth phase of experiments.

As a proxy of iron-stress, maximum photochemical quantum efficiency of photosystem II (F_v/F_m) of cells in exponential growth phase were measured using a Fluorescence Induction Relaxation System (FIRe) (SAtlantic) set to measure single-turnover flash of PSII reaction centers with a total of 50 iterations [3]. A subsample (3 mL) of the culture was dark acclimated

at the respective culture temperatures for 25 min. A saturating pulse of blue light (450 nm) was applied to low-light acclimated cells for a duration of 80 ms using the reference excitation profile. F_v/F_m was determined using the induction and relaxation protocol.

To measure the cell volumes, cell length and width of *Karlodinium* species, preserved in Lugol's solution (n = 30 for each *Karlodinium* strain), were measured using an image-analysis software program Fiji ImageJ on images collected with an inverted light microscope [4]. The shape of *Karlodinium* was estimated to be a prolate spheroid. The cell volumes of preserved *Karlodinium* species were calculated according to the following equation: volume $=4/3\pi[(\text{cell volume} + \text{cell width})/4]^3$.

Iron, carbon and nitrogen quotas

For iron-replete medium, 13.7 nmol L^{-1} (pFe 19) of $^{55}FeCl_3$ was added along with 1,356 nmol L^{-1} of non-radio-labeled $FeCl_3$ so that ^{55}Fe was 1% of [Fe]T. For the iron-deficient medium, 12.9 nmol L^{-1} (pFe 21) of $^{55}FeCl_3$ was added so that 100% of [Fe]T was radio-labeled. In TMC Teflon vials, all iron additions were pre-mixed with EDTA (1:1), and the mixture was left to sit in the medium overnight to reach equilibrium. For these experiments, cultures in exponential phase (ca. 1,000 cells) were inoculated into 28 mL PC tubes as described previously. To ensure that more than 99% of the cells were radio-labeled, the inoculum cell density was chosen to allow for at least eight cell divisions before harvesting. Growth conditions were monitored using *in vivo* fluorescence, but the final cell concentrations were determined by cell counts using 3-mL aliquots under light microscopy as described previously. Once the cultures reached mid to late exponential phase, the cells were collected using polycarbonate filters (2-µm

pore size) and soaked for 5 min with titanium-EDTA-citrate reducing solution followed by a filtered seawater rinse to remove iron adsorbed on the cell surface, as per the method described by Hudson and Morel [5]. ^{55}Fe was then measured with a liquid scintillation counter (Beckman, LS6500). For each iron treatment, absorption of ^{55}Fe onto the filter was corrected by filtering 25 mL of cell-free medium. The blank was subtracted from each measurement.

Particulate organic carbon (POC) and nitrogen (PON), were determined using mass spectrometry. For these experiments, iron-replete and iron-limited cultures in exponential phase were inoculated into an acid-washed, milli-Q water rinsed 250-mL PC bottles as described previously. Growth rates and final cell concentrations were determined by cell counts as previously described. All sub-sampling was carried out in a positive-pressure, laminar-flow hood. Samples were gently filtered through pre-combusted GF/F filters and dried in a desiccator. Filters were sent to the Stable Isotope Facility at University of California Davis to quantify the contents of POC and PON using an elemental analyzer paired with an isotope ratio mass spectrometer (EA-IRMS). Filters were combusted at 1080°C, and residual gases were passed over reduced copper and through water traps prior to gas separation and quantification via IRMS. Calibration and normalization were performed against international and in-house certified reference materials (e.g., USGS40, USGS41, Glutathione, Glutamic Acid). Limits of quantification, based on peak area, for POC and PON are 100 µg C and 20 µg N, respectively.

Mixotrophic ability

For experiment one, a dense culture of each *Karlodinium* strain maintained in standard Aquil medium and growing photosynthetically was used. Subaliquots from the cultures were

collected and fixed with Lugol's solution to determine cell concentrations. At a density of each *Karlodinium* strain as similar as possible among experiments, the potential mixotrophic growth rate of each population was compared with the growth in cultures without prey cells, by measuring the difference in cell density over the 2 days incubation. Considering their natural marine environments, oceanic and coastal isolates were experimented at 12ºC and 20ºC, respectively. As a potential edible prey species, cryptophyte *Rhodomonas salina* CCMP1319 and rhaphidophyte *Heterosigma akashiwo* CCMP1870, were chosen because not only have they been validated as prey of *Karlodinium* species [6, 7], but all of the strains except for *K. veneficum* RCC6139 we tested were observed to attack these algal prey cells when they were encountered. Despite testing other microalgal species to investigate potential optimal prey choices of *Karlodinium*, none of the oceanic isolates displayed observable feeding behavior. Initial cell concentrations of *Karlodinium* (approximately 3,000–5,000 cells mL^{-1}) and the target prey (approximately 50,000–80,000 cells mL^{-1} for *R. salina* and 10,000–20,000 cells mL^{-1} for *H. akashiwo*) were established using an autopipette. Five mL of Aquil medium was added to all the tubes, which were then filled to capacity with freshly filtered water and capped. Triplicate 28-mL PC experimental tubes (containing mixtures of dinoflagellates and prey) and triplicate predator control bottles (containing dinoflagellates only) were established. Specific growth rates (d^{-1}) of *Karlodinium* strains were calculated from linear regression of the natural log cell density versus time during the two days' incubation periods.

In experiment two each *Karlodinium* strain was investigated by observation of the prey-material ingested cells (i.e., the number of dinoflagellate cells containing food vacuoles in protoplasm relative to the number of all observed dinoflagellate cells). The protoplasm of more

than 100 *Karlodinium* cells for each strain was carefully examined using a light microscope at a magnification of 400x on an Olympus CKX-31 inverted microscope.

References

1. Brand LE, Guillard RR, Murphy LS. A method for the rapid and precise determination of acclimated phytoplankton reproduction rates. *J Plankton Res* 1981;**3**:193–201. https://doi.org/10.1093/plankt/3.2.193
2. Gustavs L, Schumann R, Eggert A *et al.* In vivo growth fluorometry: accuracy and limits of microalgal growth rate measurements in ecophysiological investigations. *Aquat Microbial Ecol* 2009;**55**:95–104. https://doi.org/10.3354/ame01291
3. Gorbunov MY, Falkowski PG. Fluorescence Induction and Relaxation (FIRe) technique and instrumentation for monitoring photosynthetic processes and primary production in aquatic ecosystems. In: van der Est A, Bruce D (eds.), *Proceedings of the 13th International Congress of Photosynthesis*. Montreal: Allen Press, 2005, 1029–31.
4. Schindelin J, Arganda-Carreras I, Frise E *et al.* Fiji: an open-source platform for biological-image analysis. *Nat methods* 2012;**9**:676–82. https://doi.org/10.1038/nmeth.2019
5. Hudson RJ, Morel FM. Distinguishing between extra- and intracellular iron in marine phytoplankton. *Limnol Oceanogr* 1989;**34**:1113–20. https://doi.org/10.4319/lo.1989.34.6.1113
6. Berge T, Hansen PJ, Moestrup Ø. Prey size spectrum and bioenergetics of the mixotrophic dinoflagellate *Karlodinium armiger*. *Aquat Microb Ecol* 2008;**50**:289–99. https://doi.org/10.3354/ame01166
7. Calbet A, Bertos M, Fuentes-Grünewald C *et al.* Intraspecific variability in *Karlodinium veneficum*: growth rates, mixotrophy, and lipid composition. *Harmful Algae* 2011;**10**:654–67. https://doi.org/10.1016/j.hal.2011.05.001

Supplementary Figures

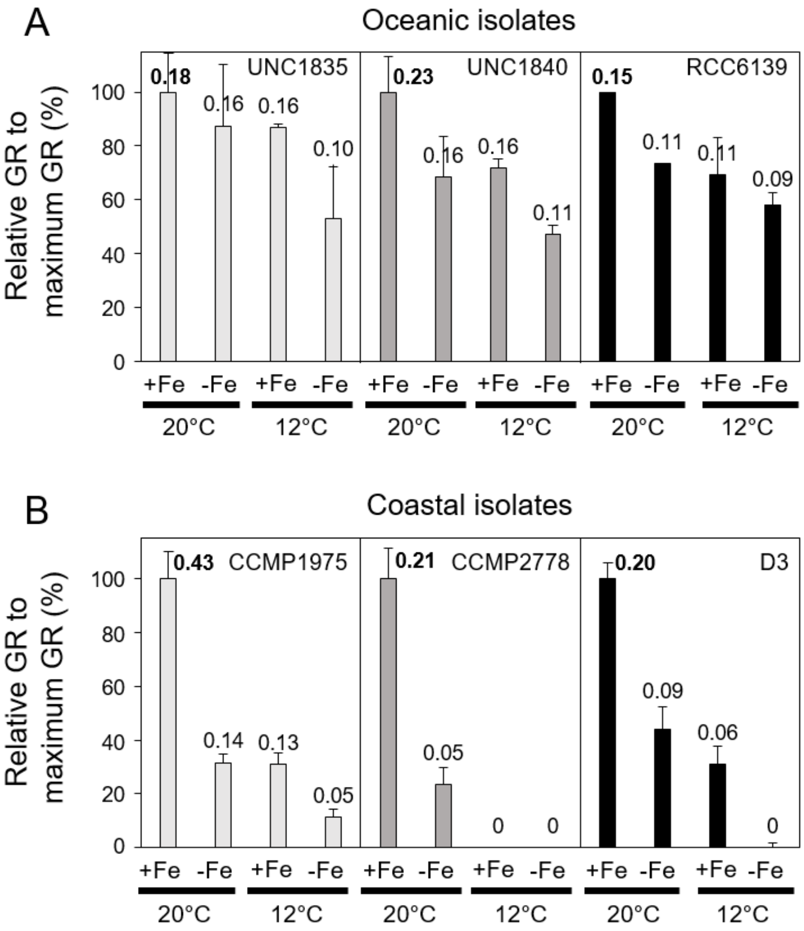

Supplementary Figure S1: Growth characteristics of six (A) oceanic (UNC1835, UNC1840, and RCC6139) and (B) coastal (CCMP1975, CCMP2778, and D3) strains of *Karlodinium* species, measured based on *in vivo* chlorophyll *a* fluorescence. The y-axis represents the relative growth rates (%) compared to the maximum growth rate under four different treatment conditions for each strain. Treatments are Fe-replete, 20ºC (+Fe, 20ºC), Fe-limited, 20ºC (-Fe, 20ºC), Fe-replete, 12ºC (+Fe, 12ºC), and Fe-limited, 12ºC (-Fe, 12ºC). The value above each bar is the specific growth rate (μ, d^{-1}) at each treatment. Error bars represent the standard deviation of biological replicates.

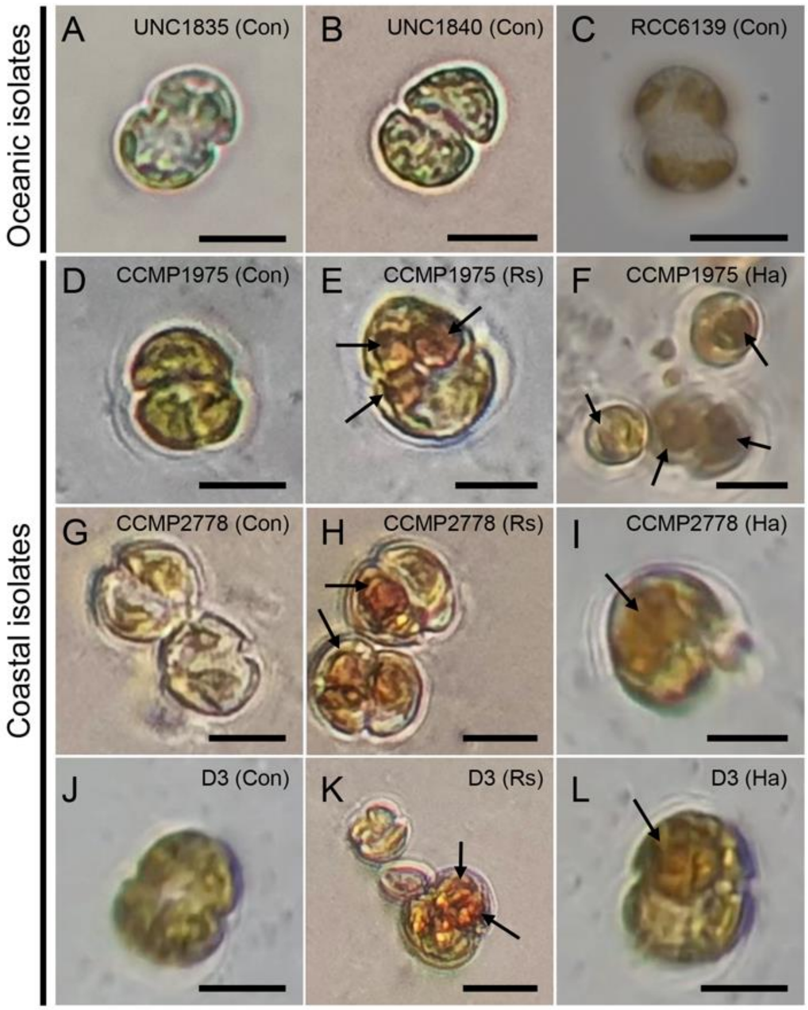

Supplementary Figure S2: Feeding by *Karlodinium* strains on prey cells: the cryptophyte *Rhodomonas salina* (Rs) (E, H, and K) and the raphidophyte *Heterosigma akashiwo* (Ha) (F, I, and L). Unfed oceanic isolates: (**A**) UNC1835, (**B**) UNC1840, and (**C**) RCC6139 (control; Con). (**D**) An unfed CCMP1975 Con cell. CCMP1975 cells (**E**) fed on Rs cells (arrows) and (**F**) fed on Ha cells (arrows). (**G**) Unfed CCMP2778 Con cells. CCMP2778 cells (**H**) fed on Rs cells (arrows) and (**I**) fed on an Ha cell (arrow). (**J**) An unfed D3 Con cell. D3 cells (**K**) fed on Rs cells (arrows) and (**L**) fed on an Ha cell (arrow). Scale bars = 10 μm.

Supplementary Table

Supplementary Table S1: List of protein-encoding genes included in Figure 4, categorized into Iron homeostasis, Photosynthesis, and Nitrogen assimilation. The table includes gene names and their corresponding functional descriptions.

Category	Gene name	Gene description
**Iron homeostasis**	*pTF*	Phytotransferrin
	ISIP3	Iron starvation-induced protein 3
	FRE	Ferric reductase
	ZIP	Iron-regulated transporter
	NRAMP	Natural resistance-associated macrophage protein
	FTN	Ferritin
**Photosynthesis**	*PETC*	Cytochrome b6f
	PETD	Cytochrome b6f complex subunit 4
	PETE	Plastocyanin (Copper-containing electron carrier)
	PETF	Fe-containing ferredoxin
	PETH	Ferredoxin–$NADP^+$ reductase
	PETJ	Cytochrome c6
	FLDA	Flavodoxin A (Iron-free alternative to ferredoxin under Fe limitation)
**Nitrogen assimilation**	*NRT*	Nitrate transporter
	NR	Nitrate reductase
	NIRB	Nitrite reductase B
	FNT	Nitrite transporter
	AMT	Ammonium transporter
	GS	Glutamine synthetase
	GLNA	GS-encoding gene
	GLT	Glutamate transporter (also known as GOGAT)
	GATase	Glutamine amidotransferase
